# Effect of *Jiawei Fengshining* on Synovial Cell Apoptosis and TGF-*β*1/Smad Signaling Pathway in Rats with Rheumatoid Arthritis

**DOI:** 10.1155/2019/8614034

**Published:** 2019-12-21

**Authors:** Xiaojun Dong, Yuan Gan, Lina Ding, Fujia Zeng, Dou Ding

**Affiliations:** Traditional Chinese Medical Department of Zunyi Medical and Pharmaceutical College, Zunyi 563006, Guizhou, China

## Abstract

**Background/Aims:**

*Jiawei Fengshining* (JWFSN) is a new formula originated from *Fengshining*, a classic formula for the treatment of rheumatoid arthritis (RA). The mechanism of JWFSN in the treatment of RA is still unclear. The aim of this study was to evaluate the effect of JWFSN formula on the inflammatory mediator levels in the serum and the TGF-*β*1/Smad pathway in the synovium and to explore the underlying mechanisms of JWFSN formula to ameliorate synovial hyperplasia and apoptosis inhibition of synovium in rats with RA.

**Method:**

SPF female Wistar rats were randomly divided into 6 groups: the blank control group, the model control group, the positive drug group, and the low-, medium-, and high- dose JWFSN groups, with 8 rats in each group. Enzyme-linked immunosorbent assay (ELISA) was used to detect inflammatory mediators, anti-inflammatory mediators, and rheumatoid factor (RF). The pathological condition and apoptosis of the synovial tissue were detected by hematoxylin and eosin (HE) and TUNEL staining, respectively. TGF-*β*1, p-Smad2, p-Smad3, and Smad7 protein expressions in synovial tissue were measured by western blot assay. In addition, human rheumatoid arthritis fibroblast-like synoviocytes cell line MH7A was treated with 20% JWFSN-containing serum to obtain *in vitro* data.

**Result:**

The administration of JWFSN was found to ameliorate synovial hyperplasia and promote apoptosis; increase the serum contents of anti-inflammatory mediators; reduce inflammatory mediators and RF contents; and inhibit the TGF-*β*1/Smad signaling pathway in CIA rats. *In vitro* JWFSN treatment increased the apoptosis of MH7A cells and decreased cell viability. Additionally, JWFSN treatment inhibited the TGF-*β*1/Smad signaling pathway in MH7A cells. Interestingly, kartogenin (TGF-*β*1/Smad pathway activator) treament reversed the effects of JWFSN treatment.

**Conclusion:**

JWFSN may ameliorate inflammatory factors' abnormality, synovial hyperplasia, and apoptosis inhibition of synovium via the TGF-*β*1/Smad signaling pathway.

## 1. Introduction

Rheumatoid arthritis (RA) is an inflammatory autoimmune disease. RA is distinguished by chronic synovitis of joints and is known to have the following pathological characteristics: synovial hyperplasia, inflammatory cell infiltration, pannus production, and damage of bones and cartilages, which finally lead to the destruction and lack of function of a whole joint [1]. This disease lasts for a long time and can also affect other organs, such as the heart, lungs, and kidney [2]. In industrialized countries, rheumatoid arthritis affects 0.5–1.0% of adults [3]. The pathogeny of RA has not yet been fully elucidated by previous researchers. However, it may be related to factors such as genetics and immune regulation [4–6]. The treatment of RA involves a combination of patient education, rest and exercise, joint protection, medications, and surgery [7]. For pharmacologic treatment, nonsteroidal anti-inflammatory drugs (NSAIDs), disease-modifying antirheumatic drugs (DMARDs), and steroid and biological response modifiers are commonly used to reduce joint pain or slow disease progression [8]. However, the application of these treatments has been limited due to the high incidence of side effects [9]. Thus, there is an urgent need to conduct further studies for novel therapies.

Transforming growth factor-beta1 (TGF-*β*1) is a multifunctional cytokine that regulates cell growth, inflammation, and angiogenesis [10]. The Smad proteins are important signal transduction molecules of TGF-*β*1. The TGF-*β*1/Smad pathway has been shown to be associated with synovial hyperplasia in RA and considered as a potential target for the treatment of RA [11–13].

*Fengshining* (FSN) is a famous Traditional Chinese Medicine prescription used for the treatment of rheumatoid arthritis, which is sourced from Mr. Bai Qingzuo, one of the four famous Chinese medicine practitioners in the Shanxi province [14]. FSN is a mixture of *Notopterygium incisum*, *Radix Angelicae Biseratae*, *Caulis Sinomenii*, *Rhizoma Wenyujin Concisum*, *Rehmannia glutinosa*, *Clematis chinensis Osbeck*, *Saposhnikovia divaricata*, *Ephedra sinica*, *Corydalis yanhusuo*, *Cyathula officinalis*, *Curcuma zedoaria*, *Rhizoma Sparganii*, *Ligusticum chuanxiong*, *Zingiber officinale*, *Glycyrrhiza uralensis*, *Cinnamomum cassia,* and *Amomum villosum*. Moreover, *Periploca forrestii Schltr* and *Gaultheria leucocarpa* var. *yunnanensis* are used in Traditional Chinese Medicine (TCM) known as “Miao medicine” and have a long history of use in the treatment of rheumatism, RA, and joint pain [15–17]. Thus, we optimized and proposed *Jiawei Fengshining* (JWFSN), based on the foundation of classic prescription “FSN” and “Miao medicine.” In this study, we aimed to investigate the potential effects of JWFSN on TGF-*β*1/Smad signaling pathway *in vivo* and *in vitro*.

## 2. Materials and Methods

### 2.1. Preparation of JWFSN

The JWFSN is an aqueous preparation from 19 medicinal herbs: *Notopterygium incisum* 18 g, *Radix Angelicae Biseratae* 18 g, *Caulis Sinomenii* 15 g, *Rhizoma Wenyujin Concisum* 15 g, *Rehmannia glutinosa* 15 g, *clematis chinensis Osbeck* 12 g, *Saposhnikovia divaricata* 12 g, *Ephedra sinica* 12 g, *Corydalis yanhusuo* 12 g, *Cyathula officinalis* 12 g, *Curcuma zedoaria* 9 g, *Rhizoma Sparganii* 9 g, *Ligusticum chuanxiong* 9 g, *Zingiber officinale* 9 g, *Glycyrrhiza uralensis* 6 g, *Cinnamomum cassia* 6 g, *Amomum villosum* 6 g, *Periploca forrestii Schltr* 15 g, and *Gaultheria leucocarpa* var. *yunnanensis* 15 g. These samples were purchased from Tongrentang Pharmacy in Chengdu, Sichuan province. The raw medicinal herbs were immersed in eight-time volumes of distilled water for 30 min and then boiled for 50 min. Thereafter, five-time volumes of distilled water were added into the residue and decocted twice for 25 min. All the collected supernatants were filtered through 8 layers of gauze, condensed into an extractum by rotary evaporators.

### 2.2. CIA Rat Model

The CIA rat model has many similarities with the symptoms in human rheumatoid arthritis and is therefore widely used in RA-related *in vivo* studies [18]. Female Wistar rats (*n* = 48, weighing 190 g to 230 g) were provided by Chengdu Dashuo Experimental Animal Co., Ltd. Rats were allowed to acclimatize for at least one week before the experiment started. Bovine type II collagen (Chondrex, USA) was emulsified in complete Freund adjuvant (Sigma, USA) with the ratio of 1 : 1 to obtain a collagen emulsion. 0.2 ml of collagen emulsion was injected into the rat-tail root. Rats in the control group were injected with an equal volume of saline. At day 7, another 0.2 ml of collagen emulsion was injected into the rat-tail root avoiding the original pinholes. Rats in the control group were again injected with equal volume saline [18]. Oral treatment with JWFSN and leflunomide was started after arthritis induction and kept for 28 days. Animals were housed and acclimatized under standard rat chow diet and tap water under climate-controlled conditions (25°C, 55% humidity, 12°h light/12°h darkness). All procedures followed were in accordance with the ethical standards of Chengdu University of Traditional Chinese Medicine Animal Care and Use Committee guidelines. The study protocol was approved by the Animal Experimental Ethical Panel of Chengdu University of Traditional Chinese Medicine.

### 2.3. Drug Administration Strategy

All the experimental rats were randomly divided into 6 groups, with 8 rats in each group. Group I: the blank control group, receiving saline treatment; group II: the model control group, suffering CIA and received no treatment; group III: the positive drug group, where CIA rats were treated with 2.33 mg/kg leflunomide (Huitian Bio-Pharma Co., Ltd., Fujian, China); group IV: the low-dose JWFSN group, where CIA rats were treated with 9.12 g/kg JWFSN; group V: the medium-dose JWFSN group, where CIA rats were treated with 18.24 g/kg JWFSN, and group VI: the high-dose JWFSN group, where CIA rats were treated with 36.48 g/kg JWFSN.

### 2.4. Preparation of JWFSN-Containing Serum

Rats were divided into two groups: negative control group and JWFSN treament group, with 10 rats in each group. Rats in the JWFSN treament group were treated with 182.4 g/kg/d JWFSN for 3 days. Rats in the negative control group were treated with an equivalent volume of normal saline (NS). After 3 days of treatment, blood was collected from the eyeball and centrifuged to gain the serum. Then, the serum was disinfected using the 0.22 *μ*m filter membrane and inactivated at 56°C for 30 min, and finally stored in the ultralow temperature freezer (Thermo, USA).

### 2.5. Cell Culture and Treatment

Human rheumatoid arthritis fibroblast-like synoviocyte cell line MH7A was obtained from Procell Life Science & Technology Co., Ltd. (Wuhan, China). MH7A cells were cultured in the DMEM/high glucose medium (Hyclone, USA) supplemented with 10% fetal bovine serum (FBS; Gibco, USA) and 1% penicillin/streptomycin (Sigma, USA). Cells were incubated in the cell incubator (Thermo, USA) at 37°C under 5% CO_2_.

Cells were divided into 4 groups. The blank control cells (BC) were cultured as normal. The negative control cells (NC) were cultured in the medium containing 20% normal serum. The JWFSN-treament cells (JWFSN) were cultured in the medium with 20% JWFSN-containing serum. The kartogenin + JWFSN treament cells (KGN + JWFSN) were pretreated using 10^−6^ M kartogenin [19] for 2 h and then cultured in the medium with 20% JWFSN-containing serum.

### 2.6. Enzyme-Linked Immunosorbent Assay (ELISA)

The rat serum rheumatoid factor (RF), interferon-*γ* (IFN-*γ*), interleukin-1*β* (IL-1*β*), interleukin-4 (IL-4), interleukin-10 (IL-10), and tumor necrosis factor-*α* (TNF-*α*) were measured using ELISA kits (Jianglai Biological Co., Ltd., Shanghai, China).

### 2.7. Histopathologic Examination

Rats were sacrificed at day 28 after arthritis induction. The synovial tissues of ankle and knee joints were taken out immediately and fixed with 4% paraformaldehyde for 3 d, decalcified with 5% formic acid, and 5 *μ*m thick paraffin sections were prepared. The pathological changes of tissues were observed under light microscope following hematoxylin and eosin (HE) staining. The apoptotic cells were stained using a TUNEL assay kit (Roche Applied Science, Germany). Images were captured with a microscope (Olympus Corporation, Japan).

### 2.8. Western Blot Assay

Protein was extracted from the synovial tissues and MH7A cells using a protein extraction kit (Boster, Wuhan, China) and quantified with the BCA kit (Thermo Fisher Scientific, USA). Equal amounts of protein were mixed with the loading buffer, boiled for 15 min at 95°C, and loaded into a 10% SDS-PAGE gel. After resolving the proteins by SDS-PAGE, the bands were transferred to PVDF membranes. Then, they were blocked for 1 h with 5% skimmed milk powder at room temperature and incubated overnight with primary antibodies against p-Smad2 (ab53100, Abcam, UK), p-Smad3 (ab63403, Abcam, UK), Smad7(ab216428, Abcam, UK), TGF-*β*1 (ab92486, Abcam, UK), and *β-*actin (ab8226, Abcam, UK). The blots were washed and incubated with goat anti-rabbit IgG H&L (ab205718, Abcam, UK) secondary antibody. The protein bands were visualized using an ECL chemiluminescence kit (EMD Millipore, Germany) and quantified using Image-ProPlus 6.0 software (Media Cybernetics, Inc., Rockville, MD, USA).

### 2.9. Immunohistochemistry

Paraffin-embedded synovial tissue sections were dewaxed through an ethanol gradient and incubated with 3% hydrogen peroxide in methanol for 10 min at room temperature to quench endogenous peroxidases and then boiled in 0.01 M sodium citrate buffer (pH = 6) for antigen retrieval, washed with PBS, and blocked with 10% normal goat serum (Thermo Fisher Scientific, USA) for 20 min at 37°C. The sections were incubated overnight with primary antibodies against XIAP (ab2541, Abcam, UK), Bcl-2 (ab59348, Abcam, UK), and Bax (ab32503, Abcam, UK) at 4°C and then incubated with the goat anti-rabbit IgG H&L (HRP polymer) (ab 214880, Abcam, UK) secondary antibody for 30 min at 37°C. After immersing in DAB (Wuhan Bioswamp Biological Co., Ltd., China) for coloration, the sections were rinsed with distilled water, counterstained with hematoxylin, dehydrated, and mounted. The stained sections were observed under a microscope (Olympus Corporation, Japan). The integrated optical density (IOD) was quantified using Image-ProPlus 6.0 software (Media Cybernetics, Inc., Rockville, MD, USA), and the mean optical density (MOD) of each sample was calculated.

### 2.10. Cell Proliferation Assay

Cell Counting Kit-8 (CCK-8) (Dojindo, Japan) assay was used to measure cell proliferation. MH7A cells in the logarithmic phase of growth were seeded into 96-well plates (6 × 10^3^/well). Cells were incubated with 10% CCK-8 solution for 1 h, and the absorbance of cells at 450 nm was measured using a microplate reader after incubation.

### 2.11. Apoptosis Assay

Annexin V-APC/PI staining and flow cytometry (FCM) were performed to detect apoptosis of MH7A cells. In brief, cells were double-labeled with Annexin V-APC/PI (Abnova, China) for 15 min at room temperature in the dark. Then, FACSCalibur™ Flow Cytometer flow cytometry (BD Biosciences, USA) was used to analyze the cell apoptosis within 1 h.

### 2.12. Statistical Analysis

Statistical analyses were performed using SPSS 20.0 software (IBM Corp., USA). Data were presented as the mean ± standard deviation (SD). One-way analysis of variance (ANOVA) was used to compare differences among multiple groups, followed by LSD's post hoc test. *P* values <0.05 were considered statistically significant.

## 3. Results

### 3.1. JWFSN Alleviated Joint Swelling in RA Rats

**Figure 1:**
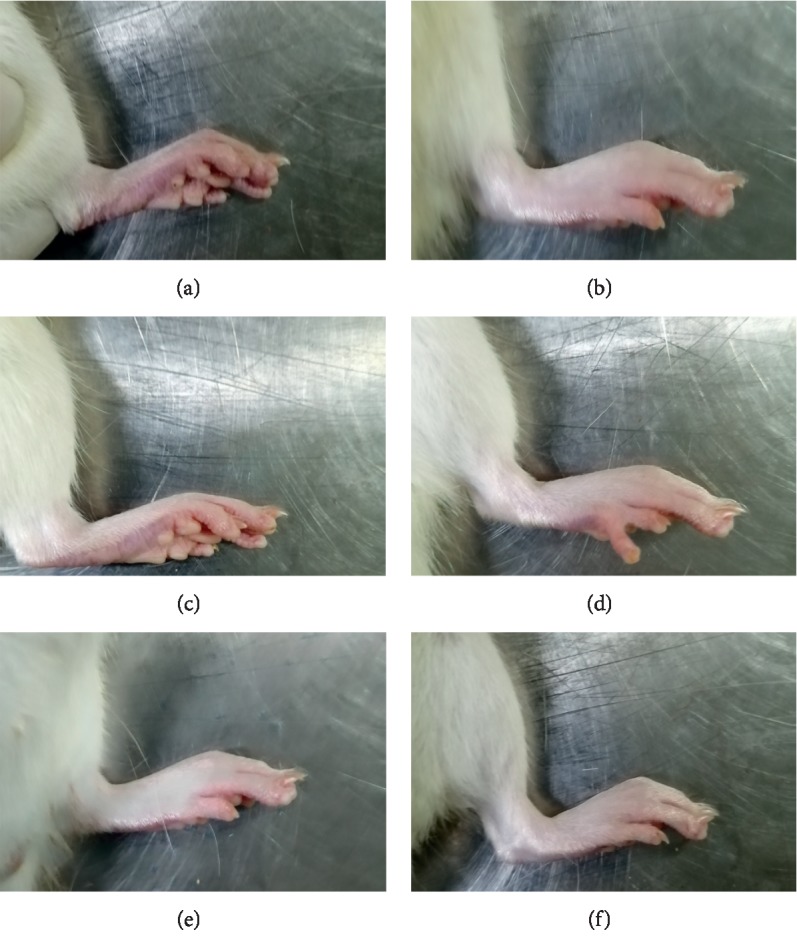
The effect of JWFSN on joint swelling in RA rats. (a) Control. (b) Model. (c) Positive. (d) Low. (e) High. Control: the control group; model: the model group; positive: the positive drug group; low: the low-dose JWFSN group; medium: the medium-dose JWFSN group; high: the high-dose JWFSN group.

As shown in Figure 1, there was hardly any swelling in the control group. However, joint swelling was observed clearly in the model group. The level of joint swelling was alleviated in JWFSN and leflunomide treatment rats compared with the model group.

### 3.2. JWFSN Restored Abnormal Changes of Inflammatory Mediators, Anti-Inflammatory Mediators, and Rheumatoid Factor in Serum

**Figure 2:**
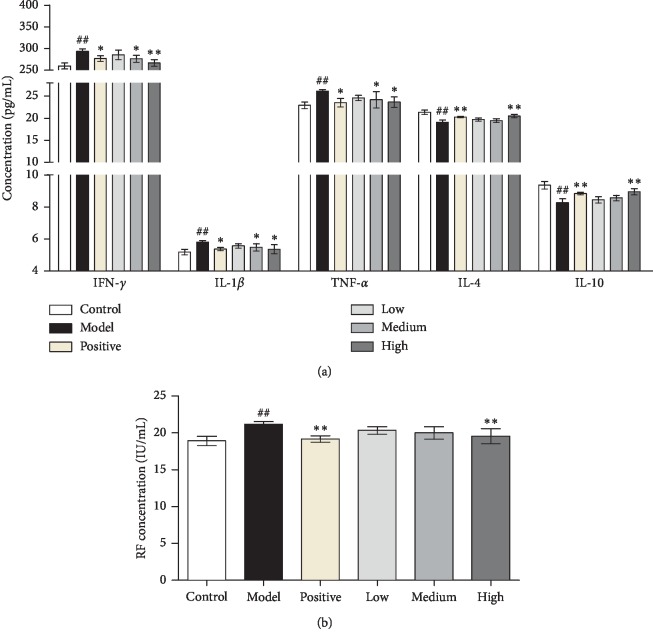
The effect of JWFSN on serum IFN-*γ*, IL-1*β*, TNF-*α*, IL-4, IL-10, and RF levels. Levels of (a) inflammatory and anti-inflammatory mediators and (b) RF in each group. The results were presented as the mean ± standard deviation. ^#^*P* < 0.05 and ^##^*P* < 0.01, compared with the control group. ^*∗*^*P* < 0.05 and ^*∗∗*^*P* < 0.01, compared with the model group. Control: the control group; model: the model group; positive: the positive drug group; low: the low-dose JWFSN group; medium: the medium-dose JWFSN group; high: the high-dose JWFSN group.

To observe the effect of JWFSN on production of inflammatory mediators, anti-inflammatory mediators, and rheumatoid factor, we examined the expression levels of INF-*γ*, IL-1*β*, TNF-*α*, IL-4, IL-10, and RF in serum of CIA rats. As shown in Figure 2, the levels of INF-*γ*, IL-1*β*, TNF-*α*, and RF in the model group was significantly higher than those in the control group. However, compared with those in the model group, the levels of the abovementioned inflammatory mediators and RF were significantly decreased in the JWFSN-high, positive drug, and JWFSN-medium groups. The levels of IL-4 and IL-10 in the model group were significantly lower than those in the control group. Compared with those in the model group, the levels of the abovementioned anti-inflammatory mediators were significantly increased in the JWFSN-high, positive drug, and JWFSN-medium groups.

### 3.3. JWFSN Restored Pathological Changes in Synovial Tissue

**Figure 3:**
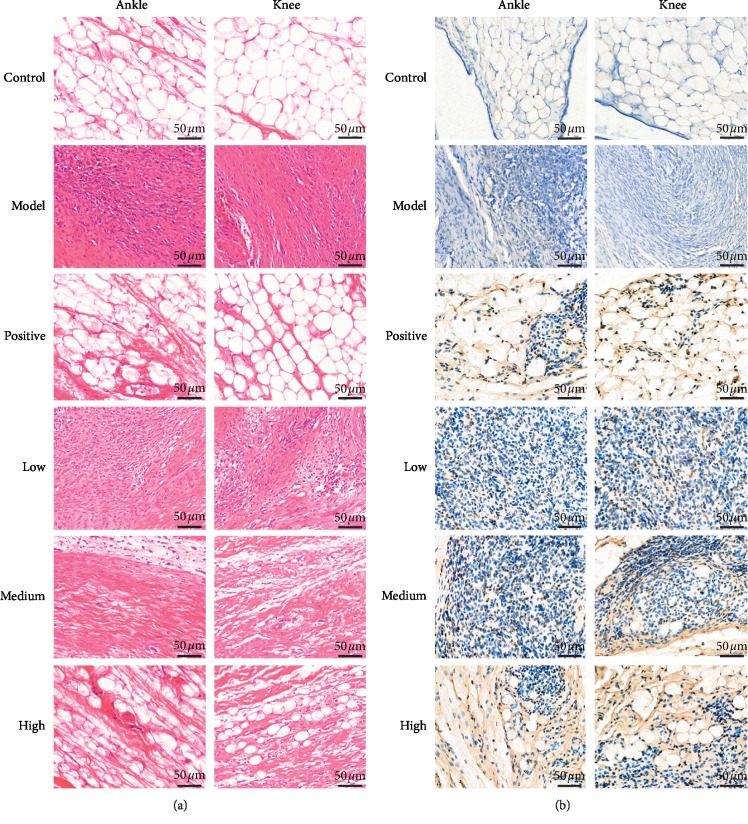
Pathological changes of synovial tissue of ankle and knee joints in each group. (a) Images of synovial tissue following hematoxylin-eosin (H&E) staining. (b) Images of synovial tissue following TUNEL staining, nucleus of apoptotic cells were stained brown. Control: the control group; model: the model group; positive: the positive drug group; low: the low-dose JWFSN group; medium: the medium-dose JWFSN group; high: the high-dose JWFSN group.

Pathological changes in the synovial tissue were measured with HE staining (Figure 3(a)). Compared with the control and positive drug groups, the model group had inflammatory cell infiltration in the synovium as well as synovial hyperplasia. Each dose of JWFSN treatment groups improved inflammatory cell infiltration and synovial hyperplasia in a dose-dependent manner. Apoptotic cells were detected by TUNEL staining (Figure 3(b)). The TUNEL-positive cells were observed in the JWFSN-low, JWFSN-medium, JWFSN-high, and positive drug groups, and almost no TUNEL-positive cells were observed in the control and model groups.

**Figure 4:**
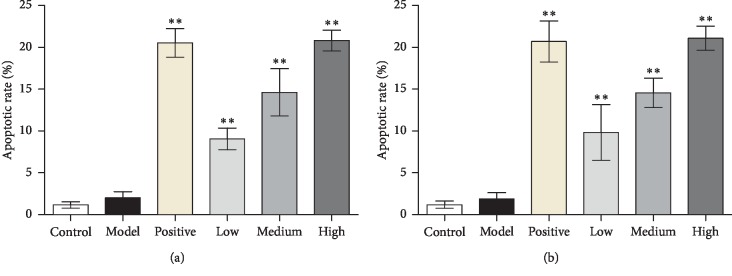
Apoptotic rate in synovium. (a) Apoptosis rate in synovium of ankle joint. (b) Apoptosis rate in synovium of knee joint. ^*∗∗*^*P* < 0.01, compared with the model group. Control: the control group; model: the model group; positive: the positive drug group; low: the low-dose JWFSN group; medium: the medium-dose JWFSN group; high: the high-dose JWFSN group.

The results of apoptotic rate in synovial tissues of ankle and knee joints indicated that there was no significant difference between the control and model groups. The apoptotic rate of JWFSN-low, JWFSN-medium, and JWFSN-high and positive drug groups were increased compared with model group (Figure 4).

These results indicated that JWFSN could improve synovial hyperplasia and promote apoptosis of synovial tissues in CIA rats.

### 3.4. JWFSN Increased Bax and Decreased XIAP and Bcl-2 Expression in Synovial Tissue

**Figure 5:**
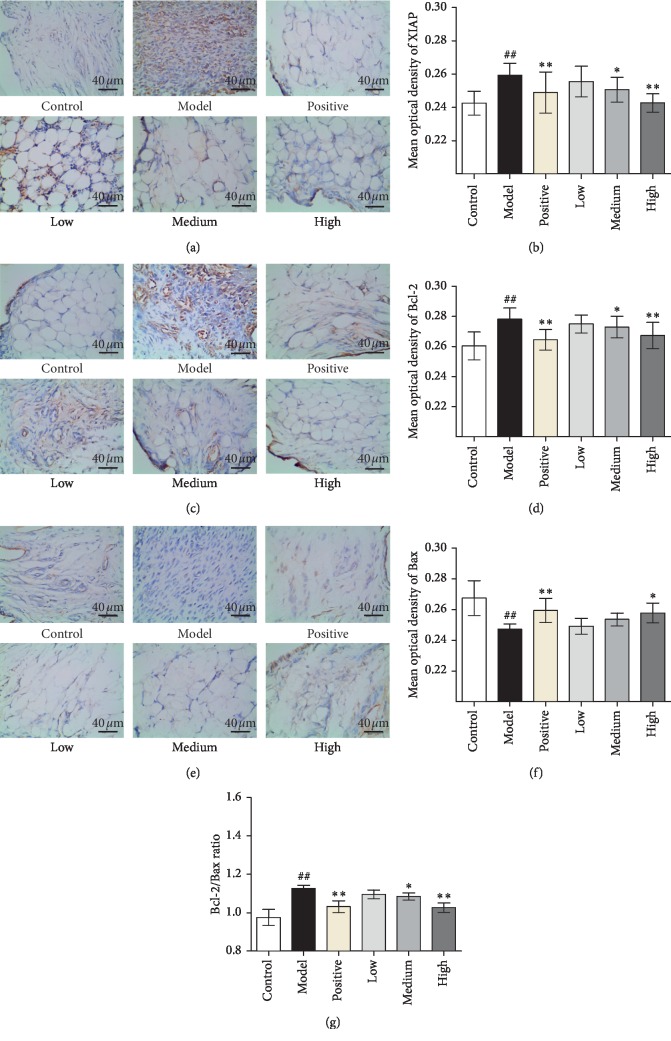
The effect of JWFSN on XIAP, Bcl-2, Bax expression in synovial tissue of ankle joint. (a) IHC for XIAP; (b) quantification of XIAP IHC staining. (c) IHC for Bcl-2; (d) quantification of Bcl-2 IHC staining. (e) IHC for Bax; (f) quantification of Bax IHC staining; (g) ratio of Bcl-2/Bax. ^#^*P* < 0.05 and ^##^*P* < 0.01, compared with the control group. ^*∗*^*P* < 0.05 and ^*∗∗*^*P* < 0.01, compared with the model group. Control: the control group; model: the model group; positive: the positive drug group; low: the low-dose JWFSN group; medium: the medium-dose JWFSN group; high: the high-dose JWFSN group.

**Figure 6:**
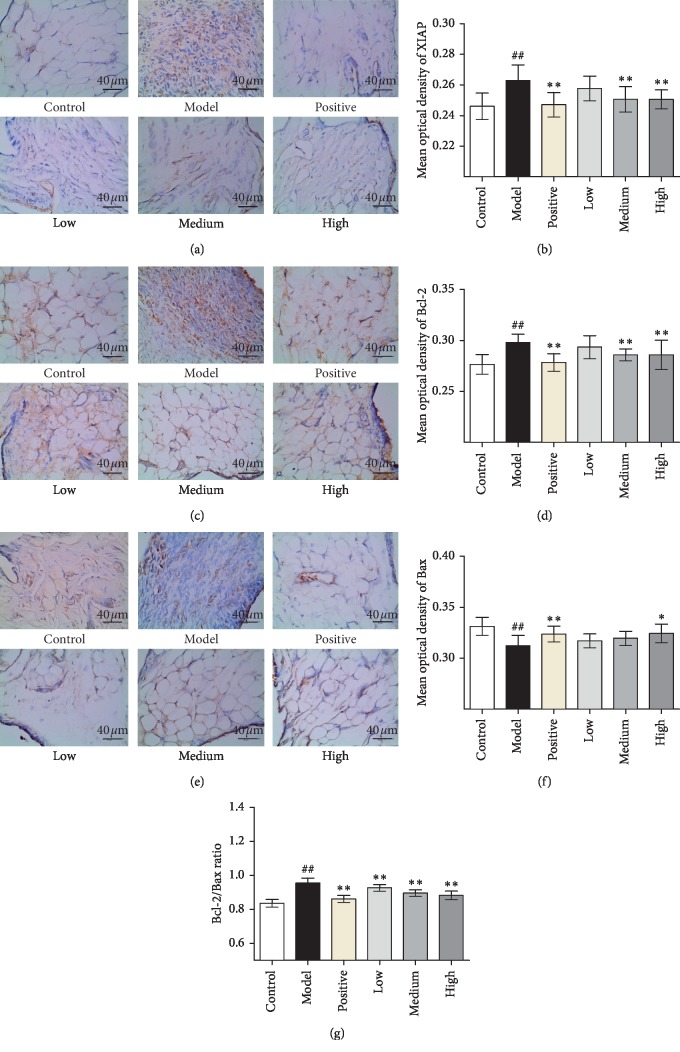
The effect of JWFSN on XIAP, Bcl-2, Bax expression in synovial tissue of knee joint. (a) IHC for XIAP; (b) quantification of XIAP IHC staining. (c) IHC for Bcl-2; (d) quantification of Bcl-2 IHC staining. (e) IHC for Bax; (f) quantification of Bax IHC staining; (g) ratio of Bcl-2/Bax. ^#^*P* < 0.05 and ^##^*P* < 0.01, compared with the control group. ^*∗*^*P* < 0.05 and ^*∗∗*^*P* < 0.01, compared with the model group. Control: the control group; model: the model group; positive: the positive drug group; low: the low-dose JWFSN group; medium: the medium-dose JWFSN group; high: the high-dose JWFSN group.

To characterize the mechanism of JWFSN-induced apoptosis in synovial tissue cells, we examined the expression levels of XIAP, Bcl-2, and Bax in ankle joints' synovial tissues of CIA rats by IHC (Figure 5). The results of IHC indicated that the expression levels of XIAP (Figure 5(b)) and Bcl-2 (Figure 5(d)) in the model group was significantly increased compared with the control group, and the expression level of Bax (Figure 5(f)) was significantly decreased. The concentration of XIAP (Figure 5(b)) and Bcl-2 (Figure 5(d)) in CIA rats was decreased in JWFSN-high and positive drug groups compared with the model group, and the expression of Bax (Figure 5(f)) was increased. The ratio of Bcl-2/Bax (Figure 5(g)) in the model group was significantly increased compared with the control group. Ratio of Bcl-2/Bax (Figure 5(g)) in JWFSN-high and positive drug groups was significantly decreased compared with the model group. The results of IHC for synovial tissues of knee joints were similar to synovial tissues of ankle joints (Figure 6).

### 3.5. JWFSN Inhibited the Activiation of TGF-*β*1/Smad Pathway in Synovial Tissue

**Figure 7:**
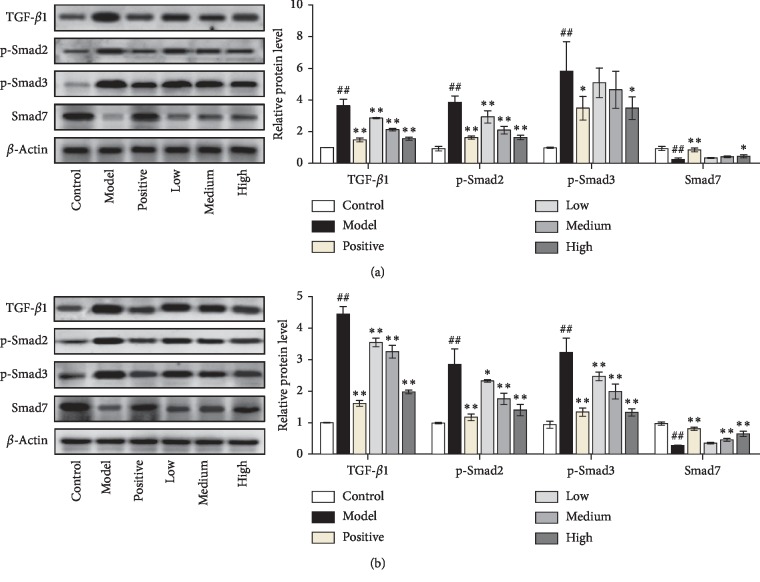
TGF-*β*1, p-Smad2, p-Smad3, and Smad7 protein levels in synovial tissue of ankle joint and knee joint. Control: the control group; model: the model group; positive: the positive drug group; low: the low-dose JWFSN group; medium: the medium-dose JWFSN group; high: the high-dose JWFSN group. ^#^*P* < 0.05 and ^##^*P* < 0.01, compared with the control group. ^*∗*^*P* < 0.05 and ^*∗∗*^*P* < 0.01, compared with the model group.

As shown in Figure 7, compared with the control group, the expression levels of TGF-*β*1, p-Smad2, and p-Smad3 in synovial tissue of ankle joint and knee joint in the model group were significantly increased, and the expression level of Smad7 was significantly decreased. Compared with model group, TGF-*β*1, p-Smad2, and p-Smad3 levels of ankle joint and knee joint synovial tissue of JWFSN-high and positive drug groups were significantly decreased, and the expression level of Smad7 was significantly increased. This result indicated that the TGF-*β*1/Smad signaling pathway was overactivated in RA rats, and the high concentration of JWFSN could reverse.

### 3.6. JWFSN Inhibited the Activiation of TGF-*β*1/Smad Pathway in MH7A Cells

**Figure 8:**
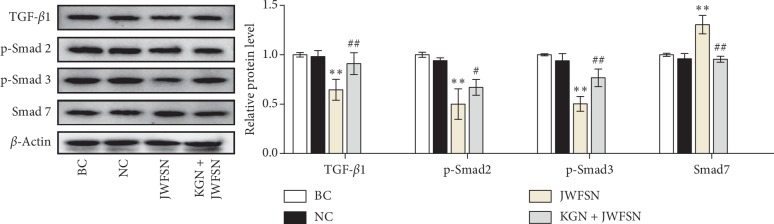
The effect of JWFSN on the expression of TGF-*β*1, p-Smad2, p-Smad3, and Smad7 in MH7A cells. BC: the blank control group; NC: the negative control group; JWFSN: the JWFSN treament group; KGN + JWFSN: the kartogenin + JWFSN treament group. ^*∗∗*^*P* < 0.01, compared with NC. ^#^*P* < 0.05 and ^##^*P* < 0.01, compared with JWFSN.

To further testify the signal transduction pathway of JWFSN, western blot assay was performed to detect the expression of TGF-*β*1, p-Smad2, p-Smad3, and Smad7 in MH7A cells. As shown in Figure 8, there was no difference in the expression levels of TGF-*β*1, p-Smad2, p-Smad3, and Smad7 between the blank control group and the negative control group. Compared with the negative control group, the expression of TGF-*β*1, p-Smad2, and p-Smad3 in the JWFSN treatment group was significantly decreased, and the expression level of Smad7 was significantly increased. However, after pretreatment with KGN, the expression of TGF-*β*1, p-Smad2, and, p-Smad3 in the KGN + JWFSN treatment group was significantly increased, and Smad7 was significantly decreased. This result indicated that JWFSN could inhibit the activation of TGF-*β*1/Smad signaling pathway, while KGN treatment reversed the effects of JWFSN.

### 3.7. JWFSN Inhibited Cell Viability and Promoted Apoptosis in MH7A Cells while KGN Reversed These Effects

**Figure 9:**
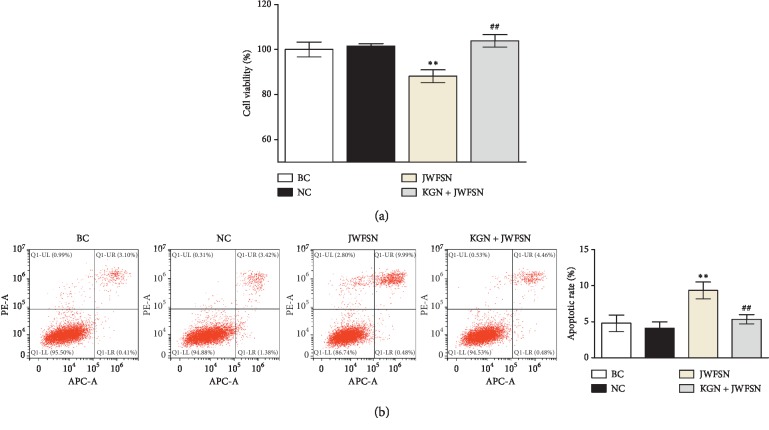
The effect of JWFSN on the cell viability and apoptosis in MH7A cells. (a) Cell viability of MH7A cells. (b) Apoptotic rate of MH7A cells. ^*∗∗*^*P* < 0.01, compared with NC. ^##^*P* < 0.01, compared with JWFSN. BC: the blank control group; NC: the negative control group; JWFSN: the JWFSN treament group; KGN + JWFSN: the kartogenin + JWFSN treament group.

To further investigate the effect of JWFSN on MH7A cells, CCK8 and FCM assays were performed. As shown in Figure 9, there was no difference in the cell viability and apoptosis between the blank control group and the negative control group. Compared with the negative control group, cell viability of the JWFSN treatment group was decreased, and apoptosis of the JWFSN treatment group was increased. After pretreatment with KGN, cell viability was increased, while apoptosis was decreased in MH7A. These data indicated that JWFSN could inhibit cell viability and promote apoptosis in MH7A cells via TGF-*β*1/Smad pathway.

## 4. Discussion

TCM-based therapies for RA are popular in China [18]. In this study, we investigated the effects of JWFSN on the pathogenesis of rheumatoid arthritis. JWFSN alleviated systemic inflammation, synovial hyperplasia, and apoptosis inhibition caused by CIA. The majority of features of CIA pathogenesis were alleviated by treatment with a high dose of JWFSN. This is the first study to report that JWFSN alleviates RA symptoms in the CIA rats.

The release of proinflammatory factors and the inhibition of anti-inflammatory factors are important characteristics of rheumatoid arthritis. Studies have found that TNF-*α*, IFN-*γ*, and IL-1*β* are significantly elevated in the peripheral blood of patients with RA [20–22]. IL-4 and IL-10 reduce joint synovial inflammation by inhibiting proinflammatory factors [23, 24]. The ELISA results of this experiment showed that the levels of serum IL-1*β* and TNF-*α* in the model group were significantly higher than those in the control group, while the levels of serum IL-4 and IL10 were significantly lower than those in the control group. The anti-inflammatory factors IL-4 and IL10 are inhibited by the increased inflammatory factors IFN-*γ*, IL-1*β*, and TNF-*α*, which proved that the CIA model prepared during the experiment was successful. Rats were given JWFSN decoction for 28 days, inhibited the expression of inflammatory factors IL-1*β* and TNF-*α*, and increased the expression of anti-inflammatory factors IL-4 and IL-10. The effect of rheumatism in the JWFSN-middle and JWFSN-high dose groups is particularly obvious, suggesting that the therapeutic effect of JWFSN on CIA rats may be through direct or indirect reduction of TNF-*α*, IFN-*γ*, and IL-1*β* expression and improve IL-4 and IL-10. It may be one of the mechanisms of rheumatoid arthritis in the treatment of rheumatoid arthritis.

Concerning H&E and TUNEL staining experiments, we found that the synovial tissues were improved inflammatory cell infiltration and synovial hyperplasia in CIA rats following JWFSN administration. Moreover, the administration of JWFSN also promoted synovial cells from apoptosis. These results indicated that JWFSN could alleviate the damage of ankle and knee joins synovial tissues. The Bcl-2 family proteins are critical regulators of the mitochondrial apoptotic pathway [25]. An increase in Bax levels or a decrease in Bcl-2 levels can trigger signals to initiate the apoptotic cascade [26]. Compared with osteoarthritis patients (OA), proapoptotic Bax was shown to be decreased, and antiapoptotic Bcl-2 was increased in FLS from RA patients [27]. Moreover, a decrease in the Bcl-2/Bax ratio has been demonstrated to promote apoptosis [28]. XIAP is an inhibitor of apoptosis protein that binds to the apoptotic promoter Caspase 9 and the effector Caspase 3 and 7 [29]. A previous study had shown that resistance to apoptosis in RA-FLS depends on the upregulation of XIAP [30]. IHC for XIAP, Bcl-2, and Bax of ankle and knee joins synovial tissues indicated that the expressions of Bcl-2 and XIAP were decreased and the expression of Bax was increased, resulting in a declined Bcl-2/Bax ratio in CIA rats following JWFSN administration. It is suggested that JWFSN promoted the apoptosis of synovial cells from CIA rats.

It is well accepted that TGF-*β*1 is mediated through both TGF-*β*R1 and TGF-*β*R2 receptors to induce phosphorylation of Smad2 and Smad3 [31–34]. Moreover, TGF-*β*1 can induce Smad7 expression, an inhibitory Smad family member, to negatively regulate TGF-*β*/Smad signaling pathway via a feedback regulation [35, 36]. Xu et al. found that abnormal activation of TGF-*β* causes articular subchondral bone destruction in the rheumatoid arthritis rat/mouse models, and conditional deletion of TGF-*β*R2 in nestin-positive cells effectively halted progression of RA joint damage [37]. Zhou et al. indicated that deletion of Smad7 promotes inflammation of rheumatoid arthritis [13]. Conversely, overexpression of Smad7 in the joint improves rheumatoid arthritis [38]. The western blot result showed that the expressions of TGF-*β*1, p-Smad2, and p-Smad3 were decreased, and the expression of Smad7 was increased in CIA rats following JWFSN administration. Additionally, JWFSN inhibited TGF-*β*1/Smad signaling pathway activity, decreased cell viability, and promoted apoptosis in MH7A cells. Interestingly, KGN reversed the effects of JWFSN treatment. These results showed that TGF-*β*1/Smad signaling pathway might be a target for the treatment of rheumatoid arthritis by JWFSN.

At the present study, we investigated the effects and the possible mechanism of JWFSN on CIA rats, with a focus on the TGF-*β*1/Smad pathway. We found that JWFSN ameliorate synovial hyperplasia and apoptosis inhibition. These effects might be associated with downregulation of TGF-*β*1, p-Smad2, and p-Smad3, and upregulation of Smad7.

## 5. Conclusion

Our results demonstrated that JWFSN inhibited synovial hyperplasia and promoted apoptosis via TGF-*β*1/Smad signaling pathway in RA. This result provides a theoretical basis for the treatment of RA by JWFSN.

## Data Availability

The datasets used or analyzed during the current study are available from the corresponding author on reasonable request.

## References

[1] Lee D. M., Weinblatt M. E. (2001). Rheumatoid arthritis. *The Lancet*.

[2] Prete M., Racanelli V., Digiglio L., Vacca A., Dammacco F., Perosa F. (2011). Extra-articular manifestations of rheumatoid arthritis: an update. *Autoimmunity Reviews*.

[3] Scott D. L., Wolfe F., Huizinga T. W. (2010). Rheumatoid arthritis. *The Lancet*.

[4] Bombardier C., Barbieri M., Parthan A. (2012). The relationship between joint damage and functional disability in rheumatoid arthritis: a systematic review. *Annals of the Rheumatic Diseases*.

[5] Lina C., Conghua W., Nan L., Ping Z. (2011). Combined treatment of etanercept and MTX reverses Th1/Th2, Th17/Treg imbalance in patients with rheumatoid arthritis. *Journal of Clinical Immunology*.

[6] Kessel A., Haj T., Peri R. (2012). Human CD19^+^ CD25^high^ B regulatory cells suppress proliferation of CD4^+^ T cells and enhance Foxp3 and CTLA-4 expression in T-regulatory cells. *Autoimmunity Reviews*.

[7] Calabrese L. H., Calabrese C., Kirchner E. (2016). The 2015 American College of rheumatology guideline for the treatment of rheumatoid arthritis should include new standards for Hepatitis B screening: comment on the article by Singh et al. *Arthritis Care & Research*.

[8] Wang C., Song Y., Wang X., Mao R., Song L. (2018). Baicalin ameliorates collagen-induced arthritis through the suppression of janus kinase 1 (JAK1)/Signal transducer and activator of transcription 3 (STAT3) signaling in mice. *Medical Science Monitor*.

[9] Feldmann M., Maini R. N. (2015). Perspectives from masters in rheumatology and autoimmunity: can we get closer to a cure for rheumatoid arthritis?. *Arthritis & Rheumatology*.

[10] Sakuma M., Hatsushika K., Koyama K. (2007). TGF-*β* type I receptor kinase inhibitor down-regulates rheumatoid synoviocytes and prevents the arthritis induced by type II collagen antibody. *International Immunology*.

[11] Cheon H., Yu S.-J., Yoo D. H., Chae I. J., Song G. G., Sohn J. (2002). Increased expression of pro-inflammatory cytokines and metalloproteinase-1 by TGF-*β*1 in synovial fibroblasts from rheumatoid arthritis and normal individuals. *Clinical & Experimental Immunology*.

[12] Geng Q., Wei Q., Wang S. (2018). Physcion 8-O-*β*-glucopyranoside extracted from Polygonum cuspidatum exhibits anti-proliferative and anti-inflammatory effects on MH7A rheumatoid arthritis-derived fibroblast-like synoviocytes through the TGF-*β*/MAPK pathway. *International Journal of Molecular Medicine*.

[13] Zhou G., Sun X., Qin Q. (2018). Loss of Smad7 promotes inflammation in rheumatoid arthritis. *Frontiers in Immunology*.

[14] Ma Y. M., Li Y. Y., Wang Y. H., Yan R. H., Chen W. C., Zhou R. (2013). Effect of fengshining capsule on reactive oxygen species-mediated T cell activation and apoptosis of synovium. *Zhongguo Zhong Xi Yi Jie He Za Zhi*.

[15] Li Y., Liu Y.-B., Yu S.-S. (2012). Cytotoxic cardenolides from the stems of Periploca forrestii. *Steroids*.

[16] Liu T., Wang X., He Y.-L. (1988). In vivo and in vitro anti-arthritic effects of cardenolide-rich and caffeoylquinic acid-rich fractions of Periploca forrestii. *Molecules (Basel, Switzerland)*.

[17] Xie M., Lu Y., Yan C. (2014). The anti-rheumatoid arthritis property of the folk medicine Dianbaizhu (Gaultheria leucocarpa var. yunnanensis, Ericaceae). *Natural Product Communications*.

[18] Wang K., Zhang D., Liu Y. (2018). Traditional Chinese medicine formula Bi-Qi capsule alleviates rheumatoid arthritis-induced inflammation, synovial hyperplasia, and cartilage destruction in rats. *Arthritis Research & Therapy*.

[19] Wang Y., Chen G., Yan J. (2018). Upregulation of SIRT1 by kartogenin enhances antioxidant functions and promotes osteogenesis in human mesenchymal stem cells. *Oxidative Medicine and Cellular Longevity*.

[20] Jeong J.-G., Kim J.-M., Cho H., Hahn W., Yu S. S., Kim S. (2004). Effects of IL-1*β* on gene expression in human rheumatoid synovial fibroblasts. *Biochemical and Biophysical Research Communications*.

[21] Brennan F. M., McInnes I. B. (2008). Evidence that cytokines play a role in rheumatoid arthritis. *Journal of Clinical Investigation*.

[22] Gao J., Kong R., Zhou X., Ji L., Zhang J., Zhao D. (2018). MiRNA-126 expression inhibits IL-23R mediated TNF-*α* or IFN-*γ* production in fibroblast-like synoviocytes in a mice model of collagen-induced rheumatoid arthritis. *Apoptosis: An International Journal on Programmed Cell Death*.

[23] Brentano F., Kyburz D., Schorr O., Gay R., Gay S. (2005). The role of toll-like receptor signalling in the pathogenesis of arthritis. *Cellular Immunology*.

[24] Barati A., Jamshidi A.-R., Ahmadi H., Aghazadeh fnm, Mirshafiey A. (2017). Effects of *β*-D-mannuronic acid, as a novel non-steroidal anti-inflammatory medication within immunosuppressive properties, on IL17, ROR*γ*t, IL4 and GATA3 gene expressions in rheumatoid arthritis patients. *Drug Design, Development and Therapy*.

[25] Liu H., Yang Y., Cai X., Gao Y., Du J., Chen S. (2015). The effects of arctigenin on human rheumatoid arthritis fibroblast-like synoviocytes. *Pharmaceutical Biology*.

[26] Luo Y., Wei Z., Chou G., Wang Z., Xia Y., Dai Y. (2014). Norisoboldine induces apoptosis of fibroblast-like synoviocytes from adjuvant-induced arthritis rats. *International Immunopharmacology*.

[27] Lee S.-Y., Kwok S.-K., Son H.-J. (2013). IL-17-mediated Bcl-2 expression regulates survival of fibroblast-like synoviocytes in rheumatoid arthritis through STAT3 activation. *Arthritis Research & Therapy*.

[28] Lončarević-Vasiljković N., Milanović D., Pešić V. (2016). Dietary restriction suppresses apoptotic cell death, promotes Bcl-2 and Bcl-xl mRNA expression and increases the Bcl-2/Bax protein ratio in the rat cortex after cortical injury. *Neurochemistry International*.

[29] Scott F. L., Denault J.-B., Riedl S. J., Shin H., Renatus M., Salvesen G. S. (2005). XIAP inhibits caspase-3 and -7 using two binding sites: evolutionarily conserved mechanism of IAPs. *The EMBO Journal*.

[30] Lattuada D., Gualtierotti R., Crotta K. (2016). Smac127 has proapoptotic and anti-inflammatory effects on rheumatoid arthritis fibroblast-like synoviocytes. *Mediators of Inflammation*.

[31] Lee Y. S., Kim J. H., Kim S. T. (2010). Smad7 and Smad6 bind to discrete regions of Pellino-1 via their MH2 domains to mediate TGF-beta1-induced negative regulation of IL-1R/TLR signaling. *Biochemical and Biophysical Research Communications*.

[32] Yang Z., Zhang X., Chen Z., Hu C. (2019). Effect of wuzi yanzong on reproductive hormones and TGF-*β*1/smads signal pathway in rats with oligoasthenozoospermia. *Evidence-Based Complementary and Alternative Medicine*.

[33] Liu J., Deng T., Wang Y. (2019). Calycosin inhibits intestinal fibrosis on CCD-18Co cells via modulating transforming growth factor-*β*/smad signaling pathway. *Pharmacology*.

[34] Oh S., Shin S., Song H., Grande J. P., Janknecht R. (2019). Relationship between ETS transcription factor ETV1 and TGF-*β*-regulated SMAD proteins in prostate cancer. *Scientific Reports*.

[35] Li J. H., Zhu H. J., Huang X. R. (2002). Smad7 inhibits fibrotic effect of TGF-*β* on renal tubular epithelial cells by blocking Smad2 activation. *Journal of the American Society of Nephrology*.

[36] Lan H. Y., Mu W., Tomita N. (2003). Inhibition of renal fibrosis by gene transfer of inducible Smad7 using ultrasound-microbubble system in rat UUO model. *Journal of the American Society of Nephrology*.

[37] Xu X., Zheng L., Bian Q. (2015). Aberrant activation of TGF-*β* in subchondral bone at the onset of rheumatoid arthritis joint destruction. *Journal of Bone and Mineral Research: The Official Journal of the American Society for Bone and Mineral Research*.

[38] Chen S.-Y., Shiau A.-L., Wu C.-L., Wang C.-R. (2016). Intraarticular overexpression of Smad7 ameliorates experimental arthritis. *Scientific Reports*.

